# Toward Super‐Resolution Reconstruction of Diffusion–Relaxation MRI Using Slice Excitation With Random Overlap (SERO)

**DOI:** 10.1002/mrm.70282

**Published:** 2026-02-01

**Authors:** Felix Mortensen, Jakub Jurek, Jens Sjölund, Geraline Vis, Ronnie Wirestam, Malwina Molendowska, Andrzej Materka, Filip Szczepankiewicz

**Affiliations:** ^1^ Department of Medical Radiation Physics Lund University Lund Sweden; ^2^ Institute of Electronics, Lodz University of Technology Łódź Poland; ^3^ Department of Information Technology Uppsala University Uppsala Sweden; ^4^ Department of Diagnostic Radiology Lund University Lund Sweden

**Keywords:** diffusion MRI, diffusion–relaxation imaging, diffusion–weighted imaging, pulse sequence design, super‐resolution reconstruction, T_1_ mapping

## Abstract

**Purpose:**

Diffusion MRI probes tissue microstructure, but low SNR and limited resolution hinder detection of features and parameter estimates. We introduce slice excitation with random overlap (SERO), which enables variable repetition times (TRs) and diffusion weighting within a single shot. This acquisition supports super‐resolution reconstruction of baseline signal ($S_{0}$), diffusivity (D), diffusional variance (V), and longitudinal relaxation ($T_{1}$) maps.

**Methods:**

We implemented a diffusion‐weighted spin‐echo sequence in Pulseq that excites thick slices at random positions. Across shots, pseudo‐random overlap produces inter‐ and intra‐slice TR variation (0.15–21.9 s) with *b*‐values up to 1.4 ms/μm^2^. The $T_{1}$‐weighting enables through‐slice super‐resolution and allows $T_{1}$ estimation. Accuracy and precision were evaluated in numerical phantoms across variable SNR. SERO was compared with slice‐shifting super‐resolution and conventional high‐resolution imaging. Feasibility was demonstrated in healthy brain in vivo at 1.5‐mm isotropic resolution in 2:30 min.

**Results:**

In simulations SERO improved accuracy of D, V, and $T_{1}$ while maintaining voxel‐wise precision comparable to direct sampling across SNRs. Regularized SERO achieved RMSE ≈ 0.5 μm^2^/ms (D) and ≈ 0.5 μm^4^/ms^2^ (V) at SNR = 3, whereas direct sampling required SNR ≥ 7–10; root‐mean–variance decreased by > 50% versus an unregularized fit. In vivo, SERO yielded sharp tissue boundaries and smooth parameter maps.

**Conclusion:**

Random slice overlap enriches encoding diversity, improving accuracy and precision of diffusion and relaxation parameters without longer scan time. SERO offers a novel path to high‐resolution microstructural imaging, especially at low SNR.

## 1 Introduction

Diffusion magnetic resonance imaging (dMRI) is an established tool for non‐invasive characterization of tissue microstructure [1]. However, dMRI suffers from an inherently low signal‐to‐noise ratio (SNR) and limited spatial resolution, both of which impede the detection of small pathological lesions [2, 3] and impact accuracy and precision of diffusion parameters [4]. Therefore, improving the trade‐off between resolution, acquisition time, and precision is valuable for clinical and research dMRI applications where characterization of subtle tissue features is of importance [5]. Several methods have successfully yielded high resolution dMRI by efficient k‐space sampling [6] and segmented readout whereby TE can be reduced [7]. Although these can be combined with image reconstruction and denoising that improves signal precision [8], they are also limited by the low SNR imposed by high‐resolution acquisitions, with a potential impact on signal accuracy [9]. As an alternative, super‐resolution reconstruction (SRR) methods have been proposed to enhance the spatial resolution of MRI while also improving the signal accuracy [10, 11]. Super‐resolution in diffusion MRI was originally explored to mitigate single‐shot EPI limits such as through‐plane blurring, geometric distortions, and coarse slice sampling [12, 13]. These methods typically employ sampling of one or more spatial dimensions at a reduced spatial resolution to boost SNR, with each low‐resolution image carrying slightly different and complementary spatial information about the underlying object [10, 14, 15]. When combined, the data can be used to estimate the corresponding high‐resolution signal or, alternatively, to directly estimate the high‐resolution parameter maps [15, 16]. SRR is complementary to segmented‐EPI with parallel imaging and compressed sensing, especially when isotropic resolution is required or through‐plane SNR is limited [11]. The fundamental challenge of super‐resolution methods is to recover information at a finer resolution than what is directly afforded by the low‐resolution images. This generally implies that the estimation is ill‐posed, that is, there exist multiple solutions with equal validity. This situation necessitates additional constraints or richer acquisitions to stabilize the reconstruction [17, 18]. Overcoming this challenge demands carefully designed acquisition and reconstruction strategies that incorporate sufficient information in the set of measurements and constraints to recover the parameters accurately.

“Slice shifting” is a technique that repeats the acquisition with thick slices acquired with a number of offsets [19]. Once data have been acquired for a given set of contiguous slices, the slices are shifted by a sub‐voxel increment, and the process is repeated. Each measurement is an average over a slightly different, overlapping, through‐plane region. SRR is permitted because every position is sampled multiple times as part of different combinations of the underlying tissue. Since this approach relies on standard pulse sequences, slice shifting is straightforward to implement and imposes no special demands on the scanner hardware or sequence execution. However, its main drawback is that the overlapping slices provide only modestly independent information, such that the inverse problem remains ill‐posed and sensitive to noise [19, 20].

Another approach is “rotating FOV imaging,” wherein conventional imaging is performed in multiple overlapping FOVs that are rotated around a common axis. This method is combined with thick slices, wherein rotation of the low‐resolution direction improves conditioning of the SRR inverse‐problem and improves through‐plane resolution compared to slice shifting [20]. However, when used with single‐shot readout techniques, which are sensitive to geometric distortions due to field inhomogeneity, the method requires a stationary phase encoding direction for each FOV rotation. Else, the object does not appear to be stationary with deleterious effects on the reconstruction. A straightforward solution is to rotate the FOV about an axis that points along the phase encoding direction, although this may have secondary drawbacks, including folding artifacts, rotation dependent shimming settings, and slice selection profiles that are difficult to trace [11, 21].

Finally, the ill‐posedness can be improved by “generalized slice dithered enhanced resolution” (gSlider) [22]. This method employs RF pulses that are tailored to excite non‐uniform profiles within one thick slab or slice. The excitations cover the same space, but each excitation introduces a complementary measurement such that a parallel‐imaging‐like reconstruction can be used to recover the high‐resolution slices [23]. Thus, gSlider can improve conditioning without rotating the FOV and offers better resolution recovery than slice shifting [22]. However, the RF pulses introduce a higher specific absorption rate (SAR), and the approach requires a specialized pulse sequence implementation. Each of the techniques summarized above addresses part of the SRR challenge, with method‐specific trade‐offs between sequence complexity, SNR efficiency, and reconstruction stability.

In this work, we aim to develop a novel method for high‐resolution imaging of diffusion and relaxation parameters without incurring longer acquisition times. We combined principles of slice shifting and variable slice excitation to yield a novel framework for SRR called “slice excitation with random overlap” (SERO). We investigated the benefits and drawbacks of SERO by numerical simulations, and in vivo experiments were performed to demonstrate the technical feasibility of SERO for brain imaging.

## 2 Methods

We detail the signal representation that underpins SERO's SRR whereafter we propose a specialized sampling scheme that exploits the unique features of SERO for SRR. Numerical simulations were used to evaluate the accuracy and precision of the estimated parameters based on SERO and reference imaging methods. Finally, a custom pulse sequence was developed to demonstrate SERO in human brain imaging.

### 2.1 Signal Forward Model

The signal forward model relates the object parameters to the measured MRI signal. In an experiment where both the repetition time (TR) and the strength of the diffusion weighting (*b*) were modulated, the signal was approximated by [24, 25]

(1) $$S\approx S_{0}\;\left(1-\exp\left(-\mathrm{TR}\cdot R_{1}\right)\right)\;\exp\left(-\mathrm{bD}+b^{2}V/2\right),$$

where the unknown parameters are the baseline signal intensity ($S_{0}$), the longitudinal relaxation rate ($R_{1}=1/T_{1}$), the apparent diffusion coefficient (D), and the diffusional variance (V) [26, 27, 28, 29].

**FIGURE 1:**
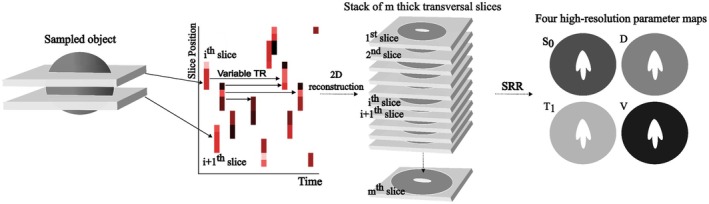
Overview of the SERO framework for data acquisition and reconstruction. For each shot, a thick slice is excited at a random location within the FOV. Since the content of each consecutively acquired slice has experienced different TRs (black to red indicates short to long TRs), a variable intra‐voxel signal $T_{1}$‐weighting, in addition to the diffusion weighting will characterize each shot. Conventional 2D image reconstruction is used to recover each $T_{1}$‐ and diffusion‐weighted thick slices, which together create a stack of all prescribed shots. Each column in the through‐slice direction of the stack provides the signal used to recover the parameter values at a high spatial resolution. Thus, the super‐resolution reconstruction is performed separately in each column of the object. The in‐plane resolution is defined by the acquired in‐plane resolution, that is, super‐resolution reconstruction is only applied in the through‐slice direction.

To generalize Equation (1) to the case of sampling with arbitrary slice excitation profiles and slice order, the signal from a single thick voxel was represented as a weighted sum of the high‐resolution voxels that it overlaps (Figure 1), with weights that describe the relative contribution from each part of the covered slice.

For simplicity, the covered slice is separated into discrete sub‐components, or sub‐voxels, defined on a high‐resolution grid that matches the target reconstruction resolution. Given the finite space in which the slices are placed, they eventually start overlapping in a random fashion such that different parts within each slice experience different TRs, that is, some parts may have just been excited (short TR) and some parts may not have been excited for a long time (long TR). This mechanism creates the heterogeneous TR within and between slices, where the local TR is determined by the time between consecutive excitations of that specific sub‐voxel (Figure 1). The variable within‐slice *T*_1_‐weighting is pivotal to improve the conditioning of the SRR (Supporting Information Section 4). The extended signal representation has spatially resolved parameter vectors that are analogues to the scalar parameters in Equation (1), according to the long TR‐approximation (see Supporting Information Section 1).

(2) $$\underset{m\times 1}{\underbrace{S}}\approx \underset{m\times n}{\underbrace{W}}⨀\underset{m\times n}{\underbrace{\left(1-\exp\left(-\mathrm{TR}⨀R_{1}^{T}\right)\right)}}⨀\underset{m\times n}{\underbrace{\exp\left(-bD^{T}+b^{⨀2}V^{T}/2\right)}}\cdot \underset{n\times 1}{\underbrace{S_{0}}},$$

where ⨀ denotes the element‐wise product, *m* is the number of shots or measurements, and *n* is the number of high‐resolution positions along the through‐slice direction. This means that each of the four parameter vectors (**S**_0_, **D**, **V**, **T**_1_) have size *n* × 1, and each element is the parameter value on the high‐resolution grid, **b** (*m* × 1) is the *b*‐value for each shot, and **TR** (*m × n*) is the position‐wise repetition time. Finally, the slice excitation profile **W** (*m* × *n*) encodes the contribution each through‐slice position has to the signal sum per shot. For example, in the case of perfectly rectangular slice selection profiles, **W** contains rows that are all zero except in clusters of ones that define the location of the thick slice on the high‐resolution grid for each given shot. Notably, the signal representation assumes accurate RF‐pulses for which it is sufficient to track only the most recent spin history in terms of the shot‐to‐shot TR. This means that $S_{0}$ is taken to be independent of earlier excitations.

### 2.2 Design of Signal Sampling Schemes

The parameters in Equation (2) can be estimated if sufficient information is acquired at each position of the object. To recover *D* and *V*, each position must be probed by at least three unique *b*‐values [27, 29]. Similarly, the longitudinal relaxation rate can be recovered if at least two TRs are used [25]. In conventional imaging methods, we vary b and TR by acquiring separate volumes with fixed TR‐*b* settings per volume. This is simple but inefficient. Unless the sequence is engineered for rapid TR changes, longer TRs insert idle time and inflate the total scan time [30]. The benefit of SERO is therefore that TR can be varied without any additional scan time penalty. The potential drawback is that the design of the sampling scheme is less straightforward as it enables vast freedom in its design. In this proof‐of‐concept, we base the sampling on uniform pseudo‐random slice positioning. Thus, the sampling density and distribution of TRs will depend on the size of the FOV relative to the slice thickness; a doubling of the FOV in the through‐slice direction will yield twice as long average TR and a two‐fold increase in the TR standard deviation. Naturally, the positioning of slice positions can be more carefully prescribed, but this was outside the scope of this work.

To compare the performance of SERO to more established methods, three sampling schemes were designed based on SERO, slice shifting, and conventional dMRI. Common among all methods was that each shot is a diffusion‐weighted spin echo with EPI readout, wherein each shot is separated by 150 ms. Under such conditions, *m* = 1000 shots were executed with an acquisition time of 2:30 min. The in‐plane resolution was 1.5 × 1.5 mm^2^. Furthermore, the FOV was 75 mm in the through‐slice direction, sampled at *n* = 50 high‐resolution positions for a targeted high‐resolution slice thickness of 1.5 mm.

#### 2.2.1 Sampling Scheme for SERO

**FIGURE 2:**
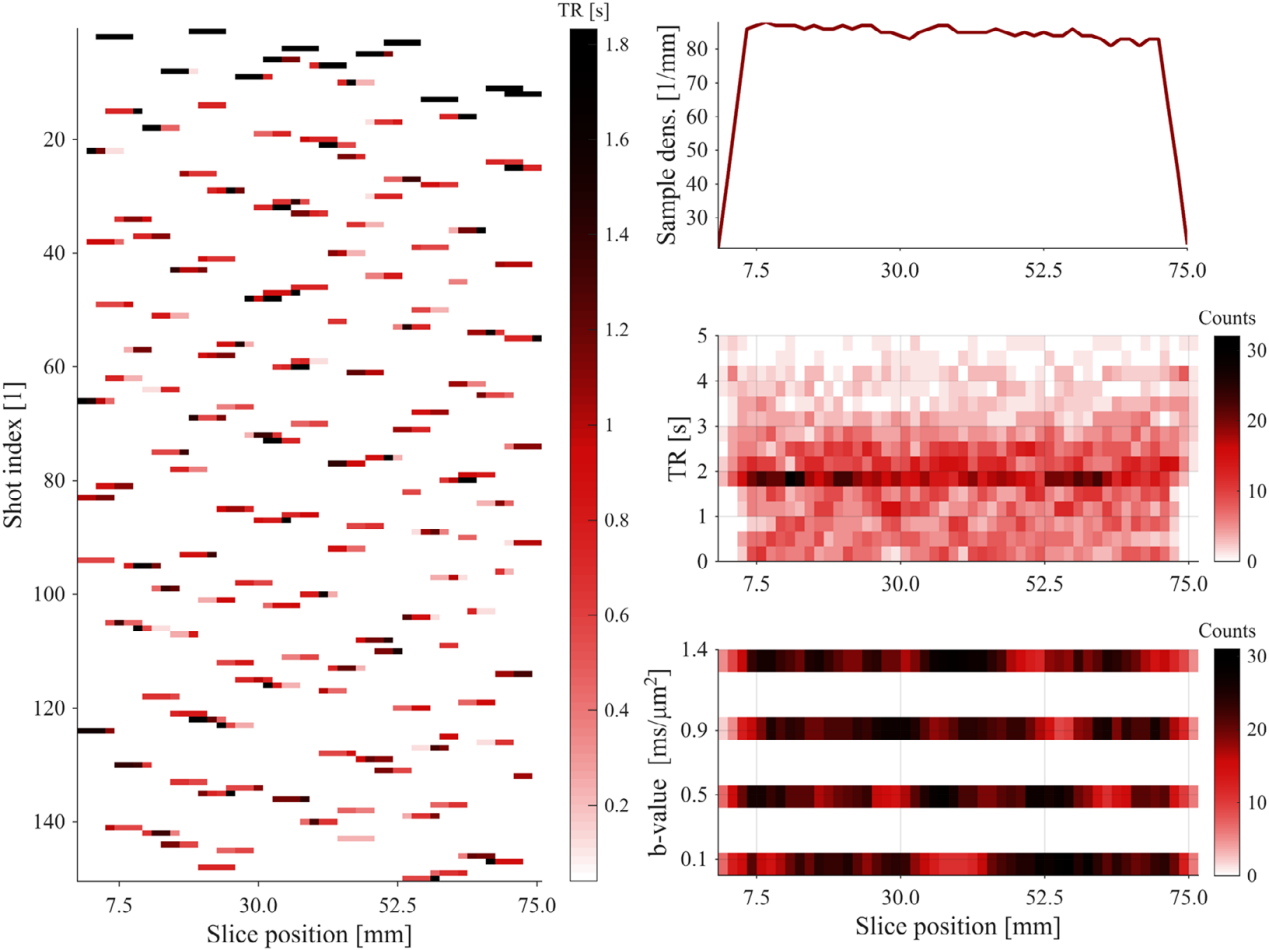
Visualization of the sampling scheme and acquisition parameters in SERO. The left panel illustrates the randomized slice excitations at the beginning of the scan (shots 1–150), with each horizontal line representing a sampled set of positions. The color of each segment shows the local TR for the specific voxel. The randomness of TR values introduces varying inter‐ and intra‐slice $T_{1}$‐weightings, enhancing the information in the data set. The sampling density plot (right panel, top row) shows the overall coverage of slice positions, with a near‐uniform distribution across most of the possible imaging positions within the FOV. However, undersampling is evident at the edges, where fewer excitations occur. The TR distribution (right panel, middle row) shows the variability in TR, with a range of TR values represented across slice positions. The *b*‐value distribution (right panel, bottom row) demonstrates that all slice positions are sampled at multiple diffusion‐weightings, with each band corresponding to one of four specific *b*‐values.

In the SERO sampling scheme the through‐slice position was randomized from a discrete uniform distribution, *z*_slice_ = [0, 1, …, *n* − *k*] · 1.5 mm, such that it excited a given set of *k* contiguous sub‐voxels that were entirely within the prescribed FOV. This process was repeated for each shot (Figure 1). If an isotropic resolution is desired, such that the through‐slice sub‐voxels have the same resolution as the in‐plane resolution, *k* can be thought of as the “aspect ratio” of the excited slices. For example, an in‐plane resolution of 1.5 mm and *k* = 4 means that the excited slice has a thickness of 6.0 mm. To reduce the frequency of slices with very short TRs that contribute low‐SNR measurements, we rejected any slice position whose slice‐averaged TR fell below 1.5 s by drawing a new randomized position. Consequently, only a small fraction of TRs falls within the lower tail of the TR distribution (< 4% of TRs are below 300 ms) whereas more than 75% of measurements have TRs above 1 s (Table 1). Finally, each shot was also assigned a random diffusion weighting where the *b*‐value was drawn from a uniform distribution of values *b* ∈ [0.1, 0.5, 0.9, 1.4] ms/μm^2^ (Figure 2). The *b*‐values were chosen to include a low‐b reference (0.1 ms/μm^2^) to minimize signal from microvasculature [31] and a high level (1.4 ms/μm^2^) to probe non‐Gaussian/diffusional variance while preserving SNR.

Using this approach, three variants of the SERO sampling scheme were produced with aspect ratios of *k*_SERO_ = 2, 4, and 6 (see Supporting Information Section 3).

#### 2.2.2 Sampling Scheme for Slice Shifting

**FIGURE 3:**
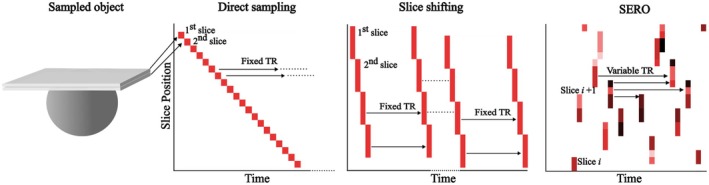
Schematic sampling schemes for conventional direct high‐resolution sampling, slice shifting, and SERO. In direct high‐resolution sampling thin, non‐overlapping 1.5‐mm slices are acquired sequentially, so every voxel is measured with the same fixed TR. In slice shifting, full volumes of 6‐mm slices are collected at a fixed TR, and then the slice center is shifted by one 1.5‐mm step, and this cycle is repeated four times, yielding complementary through‐plane positions for super‐resolution. In SERO framework, a 6‐mm slice is also excited, but positions are randomly selected on every shot, so overlapping slices assign neighboring voxels different TR histories and incidental $T_{1}$‐weighting.

For comparison, a conventional slice‐shifting protocol with aspect ratio *k*_SS_ = 4 and slice thickness of 6.0 mm was used [19] (Figure 3). To satisfy the SRR inversion criteria, four FOV positions were used, such that each FOV was shifted by 0, 1.5, 3.0 and 4.5 mm in the through‐slice direction. Each FOV position was fully acquired at a constant and uniform TR of 1.9 s, before moving on to the next FOV. To preserve steady‐state magnetization, the first two shots of every FOV were discarded from analysis; all remaining shots entered the SRR. Apart from the intentional TR schedule (variable in SERO; fixed in slice shifting), all acquisition parameters and the total scan time were matched, ensuring that observed differences reflect the encoding pattern rather than unequal acquisition times.

#### 2.2.3 Sampling Scheme for the Conventional Reference (Direct High‐Resolution)

Using the conventional reference protocol, 1.5 mm isotropic voxels (*k*_ref_ = 1) were acquired slice‐by‐slice (Figure 3), with a fixed TR of 7.5 s, using the same set of *b*‐values and acquisition parameters as the SERO and slice‐shifting scans. Capturing signal directly at the target resolution obviously removes the need for SRR. Furthermore, there are two competing effects on the signal SNR. First, the smaller voxel volume reduces the SNR by a factor of four, but the longer TR partially compensates for this loss by allowing more complete longitudinal magnetization recovery by $T_{1}$ relaxation.

### 2.3 Estimation of High‐Resolution Parameter Maps

The measured low‐resolution signal vector **S** (length m=1000) was used to estimate the four high‐resolution parameter vectors $x=\left[S_{0},D,T_{1},V\right]$ (*n* × 4 unknowns), through the forward model f(x,Θ) where Θ contains the experimental parameters (W,TR, and b). The parameter vector $\hat{x}$ was estimated by minimizing the regularized least squares objective, assuming Gaussian noise, such that

(3) $$\hat{x}=\underset{x}{\text{argmin}}{\left\|f\left(x,\Theta \right)-S\right\|}_{2}^{2}+\lambda {\left\|\Delta p\right\|}_{2}^{2},$$

where an imposed smoothness prior Δp penalizes the spatial gradient of each parameter vector, that is, the first‐order derivative in space, *λ* controls the trade‐off between data fidelity and spatial regularity (chosen through visual inspection, see Figure S1) for all parameter differences Δp, and ${\left\|\cdot \right\|}_{2}$ denotes the ℓ2 norm. Before each fit, the baseline signal $S_{0}$ was normalized by the global maximum of the measured signal to improve numerical conditioning. The optimization problem in Equation (3) was solved using a trust region method for bound‐constrained nonlinear optimization by using *lsqcurvefit* in MATLAB (The MathWorks Inc., Natick, MA, USA, version R2024a).

Initial parameter guesses $x_{0}$ were derived from a fast local fit performed slice‐by‐slice. Briefly, we obtain provisional estimates of $S_{0}$, D, V, and $T_{1}$ by fitting Equation (1) to signals observed at the position of any given sub‐voxel, assigning it the corresponding *b*‐value and slice‐averaged TR. The resulting parameter values were used as the starting point for the full SRR optimization. The parameter bounds were $S_{0}\in \left[0,4\right]\;a.u.,\;D\in \left[0.3,4\right]\;\mu m^{2}⁄\mathrm{ms},\;V\in \left[0,4\right]\;\mu m^{4}⁄{\mathrm{ms}}^{2},\;T_{1}\in \left[0.3,5\right]\;s$. Each super‐resolved reconstruction generated *n* = 50 high‐resolution parameters in a single column along the slice‐selection axis with a median fitting time of 2.6 s (inter‐quartile range was 2.2–2.9 s) using a computer with an Intel i9‐13900K 3.00 GHz CPU. The SRR was solved independently for each through‐plane column, yielding one parameter vector ${\hat{S}}_{0},\hat{D},\hat{V},{\hat{T}}_{1}$ per column. Concatenating the column solutions across the entire FOV produced the full 3D parameter maps.

Equation (3) simplifies for the direct acquisition because there is no through‐slice mixing. Each measurement excites only one through‐slice position. As a result, the estimation breaks into independent per‐voxel fits, making estimation faster. Hence, the same fitting procedures and regularization for all sampling methods were used to maintain comparability.

### 2.4 Parameter Accuracy and Precision by Numerical Simulations

To evaluate the performance of each sampling strategy under various noise and resolution conditions, two kinds of digital phantoms were used. The first kind of phantom consisted of highly variable sets of 50 × 4 high‐resolution parameter values ($S_{0},D,V,T_{1}$) designed to span a wide range of parameter combinations and to produce piecewise‐smooth through‐plane profiles with edges and plateaus. Each set of parameters represents a 1D profile along the through‐plane direction (referred to as “line phantoms”). The high variability of these objects ensures that the precision and accuracy of the methods could be evaluated for a wide variety of conditions. The second phantom was a morphologically realistic “brain phantom” available in open‐source [32]. This phantom primarily served to provide a realistic tissue geometry and a visually recognizable contrast pattern for visual inspection. Since the original phantom did not include information on diffusion, the information on diffusivity and diffusional variance was constructed from nonlinear combinations of the relaxation time maps. This procedure was guided by visual inspection of the parameter maps and resulted in realistic parameter value distributions. The resulting phantom was re‐sampled from 0.8 to 1.5 mm isotropic resolution. Both phantom types, and the code to generate them, are shared in the project data repository (see Data Availability Statement).

Regardless of the phantom type, signals were simulated by feeding the ground truth parameters into the forward model (Equation 2). To simulate the effect of noise, zero‐mean Gaussian noise was added independently to the real and imaginary channels of a signal assumed to have zero phase, with the standard deviation set to

(4) $$\sigma =\frac{\left\langle S_{0}\right\rangle }{\mathrm{SNR}},$$

where $\left\langle S_{0}\right\rangle$ refers to the mean baseline signal amplitude of the object. Taking the magnitude yields noise with a Rician distribution [9].

To place all three sampling schemes on equal footing, the SNR value in Equation (4) always refers to the isotropic high‐resolution voxel. In practice, this means that the thick‐slice methods (SERO and slice shifting) inherit a k‐fold voxel‐volume advantage, and the conventional reference protocol benefits from a longer TR on average. These differences were purposefully maintained so that the simulations mirrored the true SNR trade‐offs that each protocol would experience in real measurements. We varied SNR from 1 to 15 to span noise‐limited to high SNR regimes.

The precision and accuracy of parameters were estimated in the line phantoms by generating 50 unique parameter sets, where each was evaluated for 30 noise realizations. Accuracy was quantified by the root‐mean‐square error, $\text{RMSE}=\frac{1}{n^{2}}\sum_{i}^{n}\sqrt{{\left(x_{i}-{\hat{x}}_{i}\right)}^{2}}$, where $x_{i}$ and ${\hat{x}}_{i}$ are the true and estimated parameter values for each high‐resolution position index *i*. Precision was calculated from multiple noise realizations across identical objects as the average variance of the voxel‐wise parameters, such that the root mean variance, $\mathrm{RMV}=\sqrt{\sigma_{\text{mean}}^{2}}$. RMSE and RMV are reported for each SNR level.

To highlight the influence of spatial regularization, every reconstruction was carried out twice, that is, once with no regularization (*λ* = 0) and once with regularization *λ* = 0.01. Together, the varying SNR and the regularization analyses reveal how each acquisition strategy balances encoding diversity, voxel volume, TR and regularization strength.

The digital brain phantom was reconstructed for two SNR levels, of SNR = 30 and SNR = 10. For each noise setting, three reconstruction pipelines were compared: (i) 1.5 mm isotropic conventional imaging (Direct), (ii) SERO data fitted slice‐by‐slice without the SRR, and (iii) SERO with SRR. To facilitate a qualitative assessment of resolution fidelity, the letters “LU” were inscribed at two cranial locations in two different sizes. Through‐slice smoothing or noise‐induced artifacts would therefore manifest as blurring or loss of the inscribed region.

### 2.5 SERO Pulse Sequence Implementation and 2D‐Image Reconstruction

A custom diffusion‐weighted spin‐echo EPI pulse sequence was designed in the open‐source Pulseq framework [33]. The sequence allows user‐specified slice positions and *b*‐values for each shot, while all other functions are kept from the conventional single‐shot diffusion module. Additionally, supplementary crusher gradients were inserted after each read‐out to suppress potential spin‐history artifacts arising at short TR. To obtain a near‐rectangular slice profile, the excitation and refocusing sinc pulses were Hann‐windowed with an apodization factor of 0.3 and a time–bandwidth product of 10; the corresponding RF‐pulse duration was extended from 3 to 6 ms to satisfy hardware and safety limits. Diffusion weighting used monopolar Stejskal–Tanner gradients [24] applied along the (1, 1, 1)/√3 unit vector using synchronous gradients with peak amplitude of *G*_max_ = 49 mT/m on each axis. The sequence is shared in open source (see Data Availability Statement).

Raw Siemens TWIX data were imported with mapVBVD [34] and reconstructed offline in MATLAB with the Pulseq/Pulseq‐Recon toolkit [33]. Pulseq supplied the gradient timing and k‐space trajectory, Pulseq‐Recon handled gridding, inverse fast Fourier transform and adaptive coil combination [35], and a small modification was added to estimate N/2 ghost correction separately for each *b*‐value. The resulting 6‐mm‐thick magnitude images were then passed to the SRR algorithm to generate the final 1.5 mm isotropic parameter maps.

### 2.6 In Vivo Acquisition, Image Reconstruction, and Parameter Estimation

In vivo diffusion‐weighted images were acquired in a healthy adult male with the custom Pulseq sequence on a MAGNETOM Prisma MRI unit with a 20‐channel head coil (Siemens Healthineers, Forchheim, Germany). This study was conducted with prior approval from the Swedish Ethical Review Authority and after securing written informed consent from the participant.

Two datasets were acquired in the same volunteer: a SERO thick‐slice scan (*k*_SERO_ = 4) and a direct sampling reference at *k*_ref_ = 1. Both SERO and direct sampling used the same single‐shot spin‐echo DW‐EPI sequence, but the sampling scheme differed (Figure 3). All other sequence settings were identical. Full acquisition parameters are summarized in Table 1. In vivo SNR was estimated for both direct and SERO sampling as described in Supporting Information Section 5.

**TABLE 1:** Imaging parameters for the in vivo SERO acquisition protocol used in this study, along with the reference protocol for *m =* 1000 shots.

Parameter	SERO	Direct
FOV size (read × phase × slice)	180×180×75 mm^3^	
Acquired resolution	1.5×1.5×6.0 mm^3^	1.5×1.5×1.5 mm^3^
Target resolution	1.5×1.5×1.5 mm^3^	
Echo time	80ms	
Echo spacing	0.66ms	
Partial Fourier	6/8	
Repetition times	Min: 0.15 s First quartile: 1.12 s Median: 1.92 s Third quartile: 2.56 s Max: 21.9 s	7.5 s
Sampling density	≈80/position	20/position
*b*‐values	0.1, 0.5, 0.9, and 1.4 ms/μm^2^	
Total scan time	2:30 min	

*Note*: The parameters define the low‐resolution data set that was subsequently processed by the super‐resolution reconstruction pipeline to yield 1.5 mm isotropic diffusion–relaxation maps. For SERO, the TR distribution has an interquartile range of 1.12–2.56 s; only a small fraction (< 4%) of slice positions fall within the short‐TR tail (TR = 150 to 300 ms). Furthermore, the sampling density for SERO yields an approximate number of times the slice covers a specific position (see Figure 2), whereas for direct sampling it is exact.

## 3 Results

### 3.1 Precision and Accuracy Across Imaging Methods Using Numerical Phantoms

**FIGURE 4:**
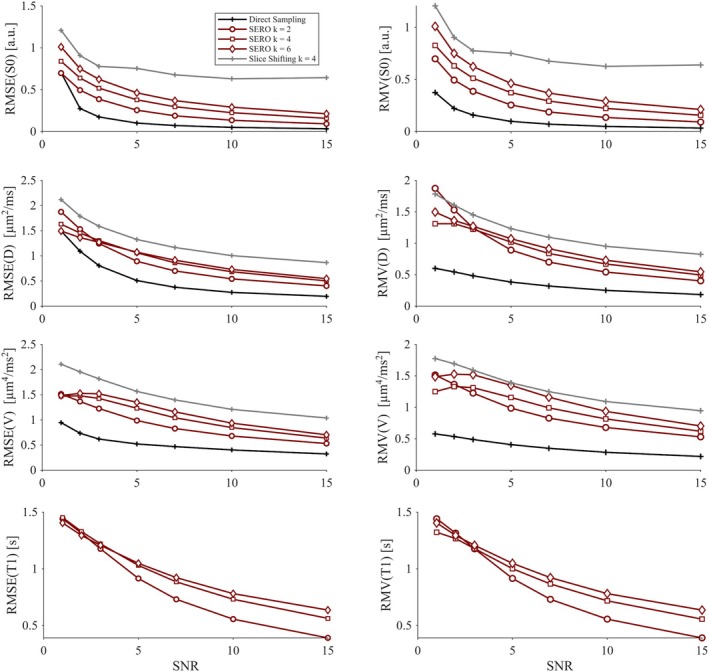
Accuracy in terms of root‐mean‐squared‐error (RMSE) and precision in terms of the root mean variance (RMV) of the unregularized estimated parameters baseline signal $S_{0}$, diffusivity D, diffusional variance V, and longitudinal relaxation time $T_{1}$ as a function of SNR for direct sampling, slice shifting, and SERO with aspect ratios, *k* = 2, 4, and 6. Direct sampling exhibited a higher accuracy and precision for all reconstructed parameters. Aspect ratio 4 lies between 2 and 6 for each parameter. The small decline in RMSE and RMV seen at the lowest SNR levels is believed to be artificial, and an unwanted effect of the inversion process.

The analysis of accuracy and precision established a clear ranking among the three sampling schemes. Without spatial regularization (*λ* = 0, Figure 4) direct sampling at 1.5 mm isotropic resolution consistently produced the highest accuracy and precision for all parameters over the entire SNR range. SERO produced intermediate errors, and slice shifting performed worst. This order persisted for every voxel aspect ratio *k*. Within SERO, accuracy and precision improved as the slices became thinner: *k* = 2 outperformed *k* = 4 and *k* = 6, and these differences decreased as SNR increased.

Accuracy decreased for all parameters as SNR was decreased. The widest spread between acquisition strategies appeared in the V parameter. At SNR = 5, the RMSE for direct sampling was approximately 0.5 μm^4^/ms^2^, 1–1.5 μm^4^/ms^2^ for SERO, and 1.5 μm^4^/ms^2^ for slice shifting. As SNR increased, both SERO and direct sampling approached the ground truth, with RMSE for $S_{0}$ approaching negligible levels already at SNR = 15. Slice shifting failed to converge even at high SNR. Overall, precision increased with higher SNR for all methods, except in the $S_{0}$ parameter for slice shifting, where it plateaued.

**FIGURE 5:**
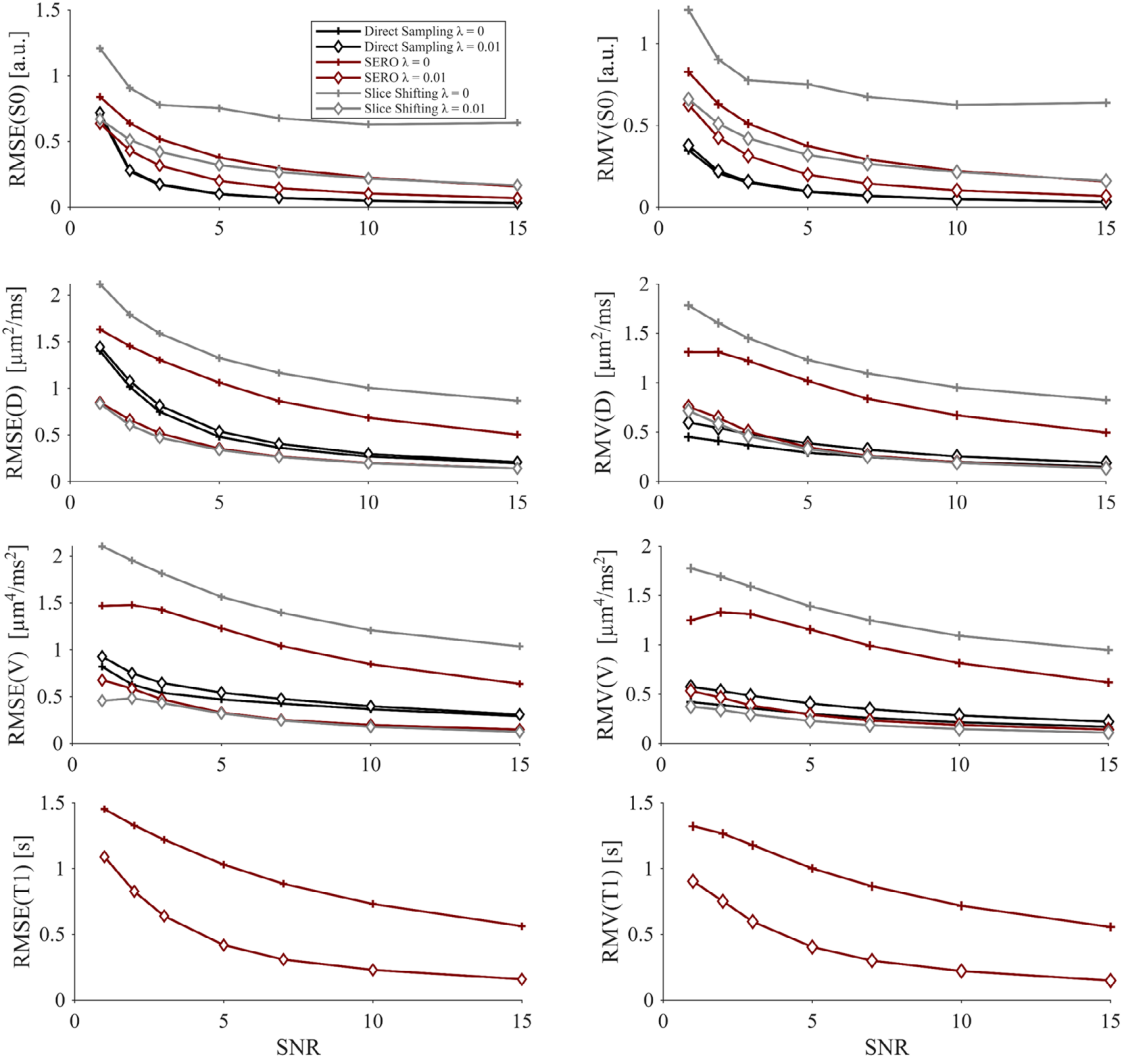
Accuracy in terms of root‐mean‐squared‐error (RMSE) and precision in terms of the root mean variance (RMV) of the estimated parameters baseline signal $S_{0}$, diffusivity D, diffusional variance V, and longitudinal relaxation time $T_{1}$ as a function of SNR (from 1 to 15) for direct sampling, slice shifting and SERO (using regularization of λ=0 and λ=0.01 and an aspect ratio *k* = 4). Direct sampling exhibited higher RMSE at low SNR for diffusivity and diffusional variance and the regularization appeared to have minimal effect, while SERO, with regularization, demonstrated a substantial bias reduction. Slice shifting converged poorly due to the ill‐posed nature of super‐resolution, resulting in elevated RMSE values. The small regularization term in SERO further improved accuracy for all parameters.

Introducing the regularization (*λ* = 0.01; see Figures 5 and S1) improved the parameter precision without reducing the accuracy. The effect was most pronounced for SERO and slice shifting, where RMSE for D,V, and $T_{1}$ decreased by more than 50% at SNR ≤ 10 and the RMV became comparable to the direct‐sampling reference. Slice shifting also showed less variability, but its ill‐posedness remained a dominant source of error for the $S_{0}$ estimation. Direct sampling was essentially unaffected by the extra smoothness constraint.

For the baseline signal, $S_{0}$, slice shifting had the worst accuracy with RMSE of approximately 0.5–0.3 a.u. across all SNR levels and showed only a modest improvement at the highest SNR. Direct sampling had the best accuracy with RMSE < 0.5 a.u. at SNR ≥ 2, while SERO had intermediate accuracy, although it also reached RMSE < 0.5 a.u. for SNR ≥ 2. The precision followed a similar pattern: direct sampling had the best precision (lowest RMV), followed by SERO, and slice shifting.

For the diffusivity, D, direct sampling had the worst accuracy at low SNR (RMSE ≈0.5 μm^2^/ms at SNR = 7) and did not match the other methods until SNR ≥ 10. SERO and slice shifting had the same accuracy already at SNR ≥ 3. By contrast, the precision was best for direct sampling at SNR ≤ 5. At higher SNR, all methods had comparable precision.

For the diffusional variance, V, direct sampling exhibited the worst accuracy (RMSE ≈0.5 μm^4^/ms^2^ at SNR = 15). SERO and slice shifting yielded lower and nearly identical accuracy across the investigated SNR levels, both showing RMSE ≈0.5 μm^4^/ms^2^ at SNR = 3 and maintaining similar variance profiles thereafter.

For $T_{1}$, which could only be recovered with SERO, the RMSE was between 1.1 and 0.2 s across all investigated SNR levels.

**FIGURE 6:**
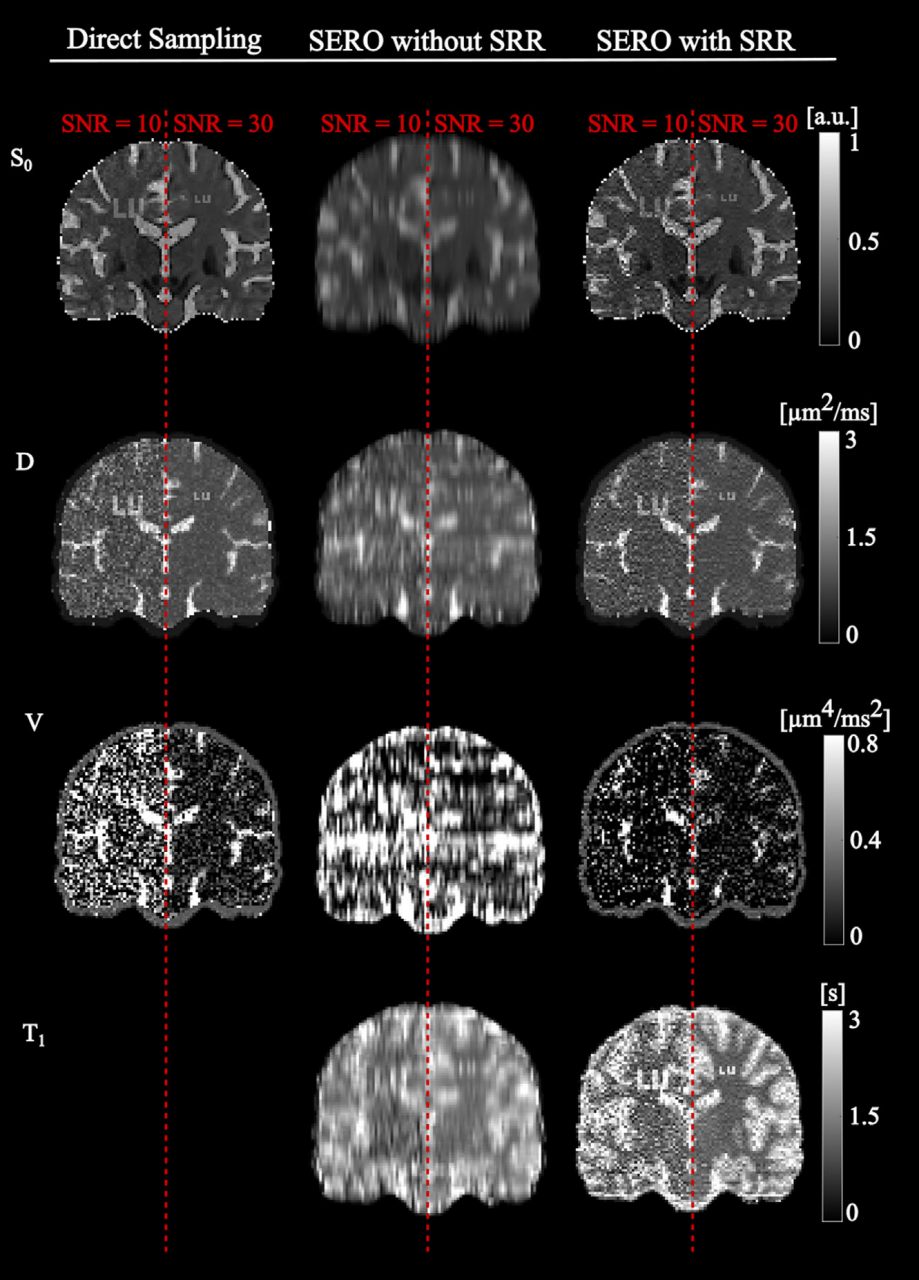
Reconstructions of the numerical brain phantom (with letters “LU” inscribed at two locations) at SNR = 10 and SNR = 30 using a regularization strength of λ=0.01, comparing direct high‐resolution sampling to the SERO framework, with a fast local parameter fit for visual comparison without SRR as well as with full SRR. The upper row shows $S_{0}$ maps, where both direct sampling and SERO produced high‐quality reconstructions with minimal noise, even at low SNR. In terms of diffusivity maps, SERO provided improved noise suppression compared to direct sampling, particularly in the case of low‐SNR. The third row shows diffusional variance maps, where SERO produced clearer reconstructions with increased robustness to noise. The red dashed vertical lines indicate the division between regions corresponding to SNR = 10 and SNR = 30. The SERO framework also enabled estimation of a robust and relatively noise‐free $T_{1}$ map, whereas no $T_{1}$ map reconstruction is available from direct sampling. SERO without SRR denotes a slice‐wise reconstruction at the 6‐mm slice resolution that fits each slice using its slice‐averaged TR (not voxel‐specific TR histories); this preserves variable‐TR contrast only at the slice level and enables $T_{1}$ estimation, but through‐plane detail is heavily blurred due to partial‐volume averaging.

Figure 6 illustrates the visual advantage of SERO with SRR compared with direct sampling. The figure displays coronal maps of $S_{0},D,$ V, and $T_{1}$ at SNR = 10 and 30, and three reconstruction settings. The $S_{0}$ maps look similar for direct sampling and SERO with SRR at both noise levels. The tissue contrast is maintained, and the LU letters are clearly legible. Differences emerge in the diffusion maps. At SNR = 10, the D map from SERO with SRR is markedly less noisy than the one from direct‐sampling, and the LU letters remain distinct; at SNR = 30 the improvement persists, although the direct scan also becomes less noisy. The benefit is even larger for V, where SERO with SRR produces sharper tissue boundaries and consistent values at both noise levels, while direct sampling is markedly corrupted by noise at SNR = 10. However, the LU letters of both sizes are lost for SERO as well as for direct sampling. Finally, SERO yielded $T_{1}$ maps wherein the SRR recovers readable LU letters at SNR = 10 and high detail at SNR 30. By comparing these results to SERO without SRR, we can visualize the nature of the underlying low‐resolution data and thereby highlight the significant contribution that SRR makes to the image fidelity.

### 3.2 In Vivo Brain Imaging

**FIGURE 7:**
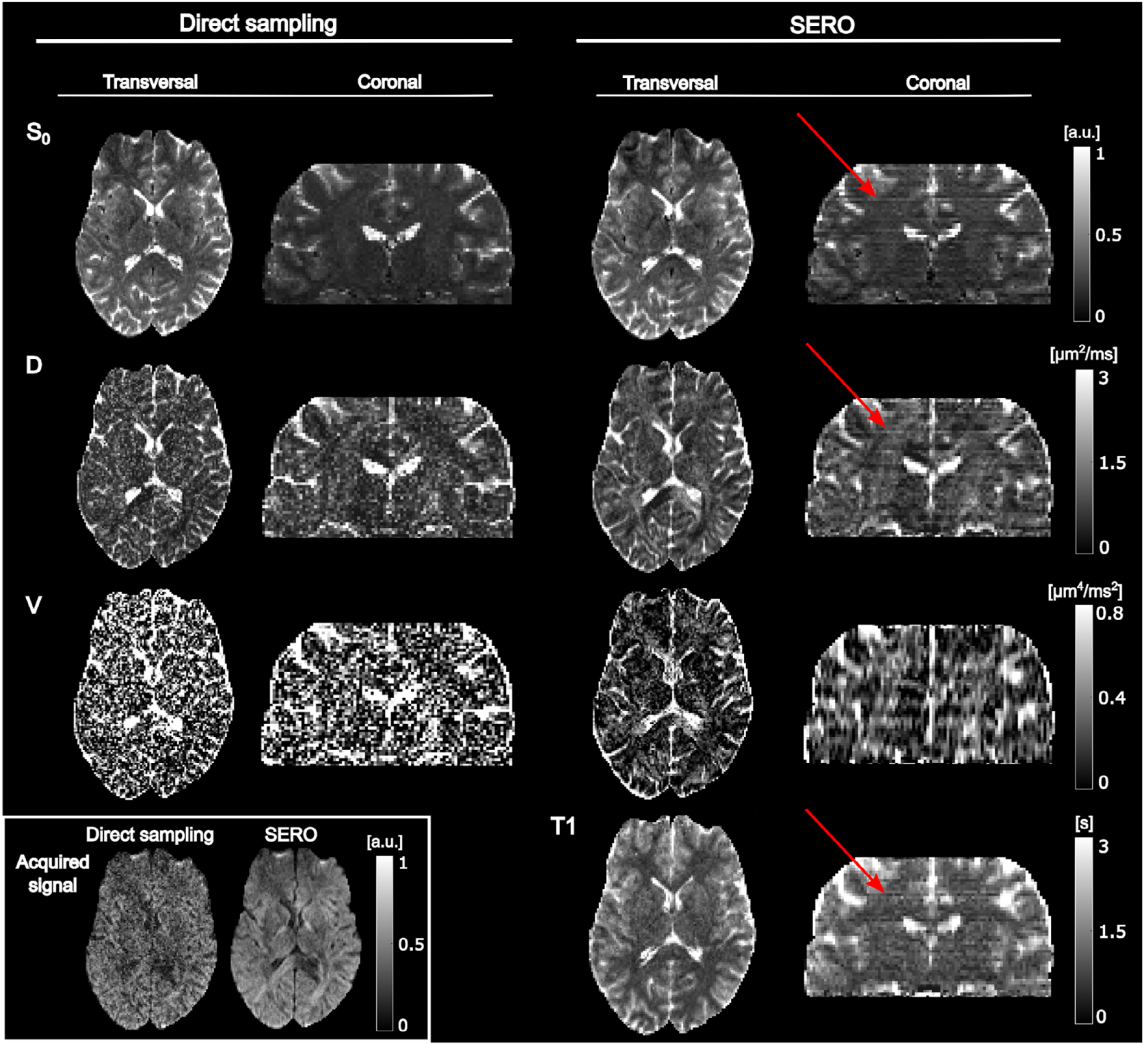
Diffusion and relaxation parameters estimated in a healthy brain, based on direct high‐resolution sampling and SERO with SRR. In the $S_{0}$ maps, both methods captured anatomical detail, yet the diffusivity image revealed noticeably lower grain and crisper tissue boundaries with SERO, indicating stronger noise suppression. The diffusional variance maps further illustrate SERO's enhanced clarity in detail and robustness to noise compared to direct sampling. Finally, the $T_{1}$ map highlights SERO's capability to produce detailed reconstructions, whereas direct sampling does not allow for reliable $T_{1}$ mapping. However, systematic line artifacts, indicated by the red arrows, were introduced in the SERO parameter maps, particularly visible in the $S_{0}$ and D maps. The lower left inset shows representative 2D reconstructed signal magnitude images from the direct 1.5 mm acquisition and the SERO 6 mm acquisition with median signal intensity inside the brain for that specific slice position (i.e., half of the magnitude images from this specific position have either lower or higher signal intensity). Reconstructions were made using λ=0.01. All data were acquired with a single diffusion‐encoding direction (*x*, *y*, *z*) = (1, 1, 1); consequently, the D and V maps are orientation‐dependent and may deviate from fully isotropic estimates. SNR levels for SERO and direct sampling were estimated to be approximately 12 and 6, respectively.

In the in vivo brain data (Figure 7), both the SERO and direct sampling methods resolved gray‐ and white‐matter. The parametric maps from SERO displayed a somewhat smoother appearance, whereas the direct‐sampling images exhibited worse parameter precision (not related to anatomy). In vivo SNR, estimated voxel‐wise from fit residuals (see Supporting Information Section 5), was approximately 12 for SERO and 6 for direct sampling.

For the baseline signal, $S_{0}$, the two methods appeared similar and showed comparable noise levels. In the D map, higher noise levels were observed from direct sampling acquisition, while the SERO sampled parameter map remained more uniform, with clearly outlined tissue boundaries. In the diffusional variance V map, spatial fluctuations from noise were more pronounced in the map from direct‐sampling method, whereas the SERO reconstruction yielded a smoother map, with clearer tissue outlines. However, the reconstruction was markedly influenced by the regularization with appreciable loss of spatial resolution in the through‐slice direction. The $T_{1}$ map from SERO showed a clear gray‐white contrast and no obvious artifacts. Generally, the appearance of parameter maps in the transversal plane had high quality without noticeable artifacts related to SRR.

Finally, we reiterate that these parameter maps were generated from a single diffusion‐encoding direction and therefore emphasize the effects of anisotropic diffusion. Thus, the diffusivity and diffusional variance maps will differ from estimates based on sampling schemes with isotropic sets of diffusion‐encoding directions and should therefore be interpreted with caution. For example, both D and V maps will vary depending on the orientation of the anisotropic tissue in the white matter.

## 4 Discussion

We have proposed a novel method for super‐resolution imaging of diffusion and relaxation parameters by exploiting slice excitation with random overlap. It was demonstrated that SERO can be used to achieve heterogeneous TRs, both within and between slices, and that the resulting T_1_‐weighting improved the estimation of high‐resolution parameter maps (see Supporting Information) at no additional cost to acquisition time. With the addition of a modest regularization, the SERO approach enables improved parameter accuracy that is on par with, or superior to high‐resolution direct sampling. Moreover, an additional value of the SERO method is that it enables simultaneous estimation of the longitudinal relaxation times within a single scan, similar to the ZEBRA sequence developed by Hutter et al. [30]. However, here the TR variation arises intrinsically from random slice overlap rather than prescribed inversion times. Taken together, SERO with SRR provides a potential improvement in the trade‐off between acquisition time, image resolution, and SNR, while producing a set of parametric maps. To promote the use and improvement of this framework, we have shared the pulse sequence as well as the code for generating sampling schemes and parameter estimation in open source (see Data Availability Statement).

Simulations showed that SERO maintained a higher accuracy for diffusivity D and diffusional variance V, compared with direct sampling, over the entire SNR range when regularization was applied (Figure 5). Translating this SNR gain into voxel size or scan time, a conventional 1.5 mm isotropic acquisition would require a four‐fold voxel volume or 16 times longer scan time to match the noise robustness that SERO achieves with 6 mm thick slices and SRR. Furthermore, the lower accuracy observed at low SNR with direct sampling is consistent with the rectified noise‐floor effect, where magnitude reconstruction causes a positive bias at low signal levels and drives parameter bias [9, 36, 37]. As shown by Vis et al. [11], this mainly causes a positive bias in diffusional variance, but can be effectively suppressed using larger voxels. Thus, the acquisition of thick slices in SERO boosts the SNR, and the associated variation in TR provides additional contrast that the super‐resolution algorithm benefits from to recover accurate high‐resolution maps [38, 39].

In contrast to D and V, the baseline signal $S_{0}$ exhibited comparable accuracy for both direct sampling and SERO. Slice shifting never attained these accuracy levels for $S_{0}$, reflecting the ill‐posedness of its sampling pattern in agreement with previous studies [10, 14, 17, 20]. These results underscore the need for enhanced encoding strategies, such as the variable‐TR framework in SERO to achieve both high resolution and robust parameter estimation.

Although SERO is better conditioned than slice shifting, it still faces the fundamental super‐resolution problem of attempting to recover a higher resolution than there is direct support for in the thick‐slice measurements [38, 39]. This means that the reconstructed parameter values can be particularly sensitive to noise. The regularization serves to suppress this sensitivity, and a moderate smoothness constraint appeared to be sufficient to stabilize SRR based on SERO, increasing both its accuracy and precision.

Qualitative evaluation of parameter maps in the brain phantom (Figure 6) was consistent with the results of the simulations. At SNR = 10, SERO preserved fine structures in the diffusivity and variance maps, whereas from the direct sampling, these were markedly more affected by noise. The LU letters were clearly recognizable in the SERO $S_{0},D$, and $T_{1}$ maps. Direct sampling produced comparable $S_{0}$ and D. The visual observations that reduced precision manifesting as higher voxel‐wise variance is the main factor that limits image interpretability [4, 36, 40] at SNR = 10 level. The improvement at SNR = 30 reflects the corresponding reduction in signal variance.

Maps of diffusivity and diffusional variance in vivo reconstructed with SERO (Figure 7) were visibly less noisy and showed sharper tissue boundaries than those obtained from the direct‐sampling scan within the same scan‐time, echoing the previous results. These in vivo findings confirm that the extra information encoded by random slice overlap and shot‐to‐shot TR variation is preserved during acquisition and can be exploited by the nonlinear reconstruction.

The smoother, sharper diffusivity and variance maps produced by SERO together with a $T_{1}$ map show that high‐resolution microstructural contrasts can be obtained within the same acquisition window of direct sampling. Similar gains in noise suppression and spatial detail have been reported for other thick‐slice super‐resolution strategies that add extra encoding diversity [10, 11, 14, 22, 23]. Whether these technical improvements translate into diagnostic benefit remains to be established, but the present results suggest that SERO may offer an alternative to conventional sampling when SNR is limited.

The present work has several important limitations. First, the parameter maps estimated in vivo exhibited horizontal stripe artifacts (Figure 7). The fact that they are present across the whole brain in all parameter maps suggests a systematic error in the estimation. Since the artifact is not seen in the digital phantoms, the artifact is likely caused by an imperfect forward model (Equation 2) in combination with the non‐homogeneous sampling scheme. This includes effects such as influence from higher order terms in the cumulant expansion [41, 42] as well as imperfections in the pulse sequence implementation or sampling scheme. For example, our signal model (Equation 2) uses the long‐TR approximation of the full expression in Equation (S1) and together with crushers, assumes that only the immediately preceding excitation affects the longitudinal magnetization. However, in practice, inaccurate flip angles may introduce dependencies on earlier excitations, which are not straightforward to track. Such effects may contribute both loss of precision and accuracy in the estimated parameters. We expect that a careful and systematic design of the sampling scheme could achieve even fewer samples at extremely short TRs as well as equivalent sampling at all positions, such that the potential bias would be similar across positions, thereby suppressing the line artifacts. Furthermore, although the $T_{1}$ map does not incur any additional scan time and is reconstructed in the same image space as the diffusion, it is not expected to have the same quality as dedicated $T_{1}$ mapping methods. Second, the nonlinear estimation is relatively slow (median fitting time of 2.6 s per 50 × 4 parameter values), and accelerating the solver or using a machine learning approach are promising venues for reducing the fitting time [43]. Third, a single, globally fixed regularization weight, chosen heuristically, was employed in the parameter estimation (Equation 3). Although this stabilized the parameter estimation, it inevitably imposed a smoothness constraint that may reduce the conspicuity of fine details. Future investigations should strive for a data‐driven regularization and to determine its potential downsides in terms of parameter accuracy and spatial resolution. An alternative to the current regularization would be to replace it with an edge‐preserving regularizer, such as total variation or total generalized variation which permits sharp transitions at tissue boundaries without over‐smoothing fine structures [44, 45]. Another option would be to employ spatially adaptive regularization, wherein a map of local weighting parameters is estimated (e.g., via the discrepancy principle) so that homogeneous regions receive stronger smoothing while fine‐detail areas are preserved [46]. Also, low‐rank and sparsity decompositions exploit global redundancies together with sparse or total variation constraints to stabilize the inversion yet retaining local detail, as demonstrated in accelerated MRI parameter mapping [47]. Fourth, the present signal model (Equation 2) represents diffusion weighting by scalar *b*‐values. Our in vivo demonstration employed a single diffusion‐encoding direction, which accentuates anisotropy and residual orientation dependence in D and V. Estimates of the directional average of D and V would require multiple directions with appropriate averaging or isotropic diffusion encoding [48, 49]. Furthermore, the signal model assumes that the effect of inversion is negligible which may introduce a slight bias, especially for sampling that is dense at short TRs (see Supporting Information Section 1). However, the full model including inversion effects is compatible with the current sampling and available in open source (see Data Availability Statement). These choices reflect the prototype nature of our implementation, whose primary aim was to demonstrate a novel approach to SRR. In future developments, we expect that the sequence and reconstruction can be made to incorporate arbitrary diffusion encoding to recover the full diffusion tensor or higher‐order parameters [27, 50, 51]. Finally, as with other single‐shot SE‐EPI methods, geometric distortion and eddy‐current effects can be mitigated post hoc; however, SERO's nonuniform per‐shot contrast and TR histories mean that off‐the‐shelf volume‐wise motion correction is not directly applicable. We therefore anticipate that robust motion handling will benefit from a TR‐history–aware, slice‐wise or joint motion‐and‐reconstruction strategy integrated into SRR. Addressing these limitations will be essential for translating SERO from a proof‐of‐concept technique into a robust research tool for larger studies.

## 5 Conclusions

This study shows that combining SERO acquisition with a regularized SRR enables diffusion–relaxation maps that have advantages in terms of accuracy and precision compared to similar methods especially when SNR is limited. By exploiting inter‐shot encoding diversity, the SERO framework lowers the SNR threshold needed for reliable parameter estimation and renders a $T_{1}$ map without the need for an extended acquisition time. We present SERO as a novel conceptual strategy that points to the possibility of achieving finer spatial detail while preserving high‐accuracy quantitative MRI without proportionally increasing acquisition cost. Future work will aim to refine the encoding schedule and reconstruction algorithms to test the method across broader settings.

## Funding

This work was supported by the Swedish Research Council (2021‐04844), the Swedish Cancer Society (22 0592 JIA, and 22 2011 Pj), the Crafoord Foundation (20240791), the Wallenberg AI, Autonomous Systems and Software Program (WASP) funded by the Knut and Alice Wallenberg Foundation, and the Swedish Prostate Cancer Federation. Jakub Jurek is supported by Grant No. 2024/08/X/ST6/01689 from National Science Centre, Poland.

## Conflicts of Interest

The authors declare no conflicts of interest.

## Supporting information


**Data S1:** This file expands the method and presents additional analyses that support the study.

## Data Availability

All codes used in this study are available at https://github.com/felixmortensen/SERO. The repository contains Pulseq functions for sequence construction and data reconstruction, super‐resolution reconstruction routines, and scripts for preprocessing and model fitting. The in vivo MRI datasets underlying the results contain potentially re‐identifiable information and are available upon reasonable request from the corresponding author.
