# Supplementary material for: Toward Super‐Resolution Reconstruction of Diffusion–Relaxation MRI Using Slice Excitation With Random Overlap (SERO)
Source: Magn Reson Med. 2026 Feb 1;95(6):3213–26. doi: 10.1002/mrm.70282 (PMC13049258; doi:10.1002/mrm.70282)
Supplement: Supplementary file 1 — Data S1: This file expands the method and presents additional analyses that support the study. [file MRM-95-3213-s001.docx]

### **1. The forward model**

In each shot, the signal magnitude is described as a slice-profile weighted sum of high-resolution voxel contributions, according to

| $\mathbf{S}=\mathbf{W}⨀\left[ 1-2\exp\left( -\left( \mathbf{TR}-TE/2 \right)⨀\mathbf{R}_{1}^{T} \right)+\exp\left( -\mathbf{TR}⨀\mathbf{R}_{1}^{T} \right) \right]$  $⨀\exp(-\mathbf{b}\mathbf{D}^{T}+\mathbf{b}^{\odot2}\mathbf{V}^{T}/2){\cdot\mathbf{S}}_{0}.$ | (Eq. S1) |
| --- | --- |

The first exponential term captures the effect of inversion, whereas the second exponential term captures longitudinal recovery between two overlapping shots. These effects are substantial when the TR is short compared to TE. When TR is long relative to TE, as is the predominant case in this study, the first term becomes small, and the model can be simplified by the approximation

| $\mathbf{S}=\mathbf{W}⨀\left[ 1-\exp(-\mathbf{TR}⨀\mathbf{R1}^{T}) \right]⨀\exp(-\mathbf{b}\mathbf{D}^{T}+\mathbf{b}^{\odot2}\mathbf{V}^{T}/2){\cdot\mathbf{S}}_{0},$ | (Eq. S2) |
| --- | --- |

as stated in Eq. 2 in the text. Note that both variants are implemented in the reconstruction code and give similar results given the employed sampling schemes.


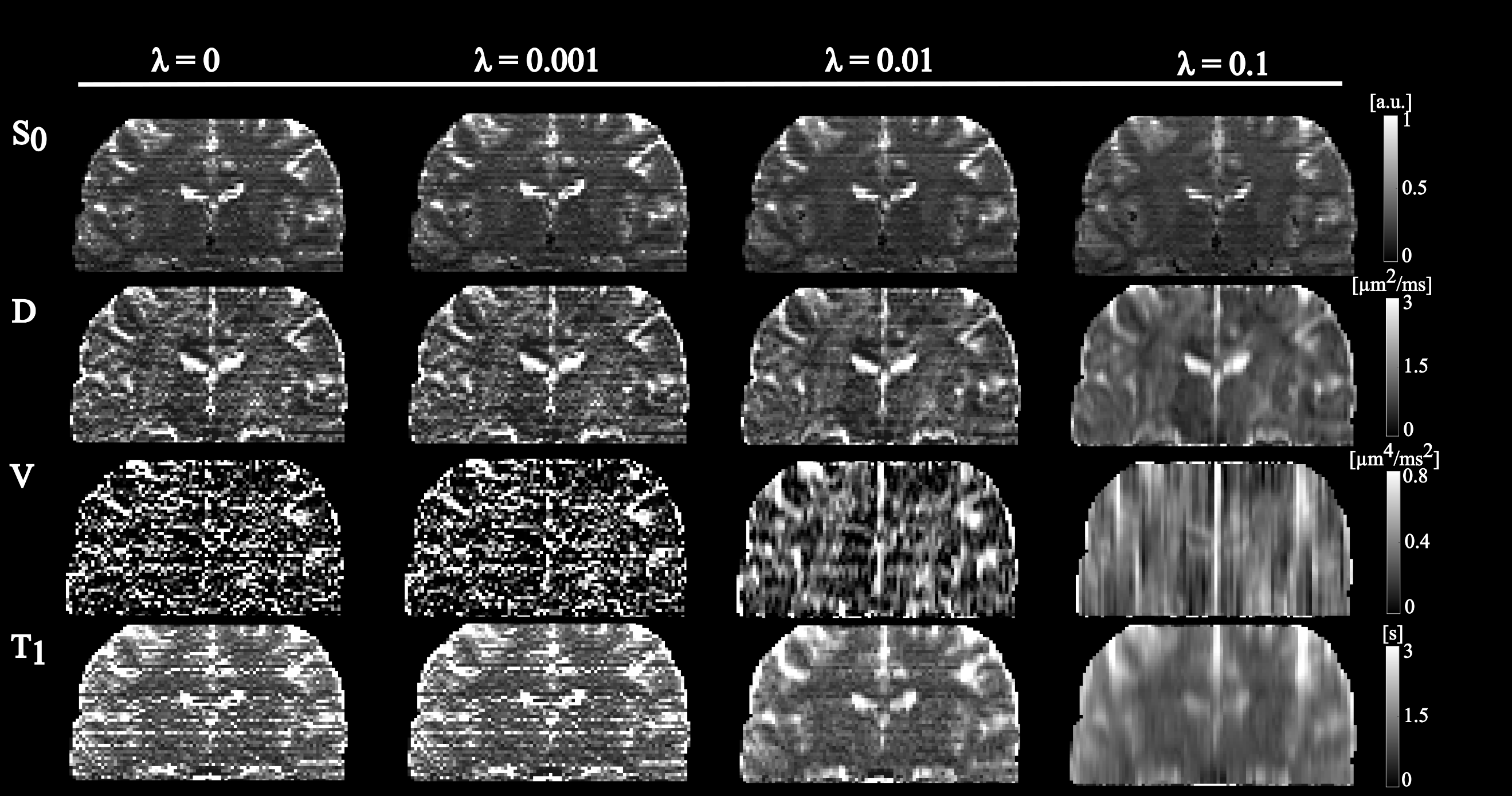


Figure S1 *–* Influence of the regularization strength (λ) on parameters reconstructions in vivo using SERO framework. Coronal slices from a healthy brain are shown for three regularization strengths. Rows correspond to the recovered parameter maps, i.e. baseline signal *S*_0_, diffusivity (D), diffusional variance (V), and longitudinal relaxation time (T_1_). Increasing λ progressively removed noise: λ = 0 is noisy, λ = 0.001 offers modest improvement, λ = 0.01 yields reduction in noise levels without obvious blurring, whereas λ = 0.1 blurs out fine details to a high extent.

### **2. Regularization strength comparison**

Figure S1 shows the effect of different regularization strengths on the reconstructed images. We tested λ = 0 (no regularization), λ = 0.001, λ = 0.01 and λ = 0.1. The images reconstructed without regularization were markedly noisy, while those with strong regularization (λ > 0.01) appeared overly smooth and lost fine details. A balanced trade-off between data fidelity and smoothness was achieved at λ ≈ 0.01.

### **3. Aspect Ratios at λ = 0.01**

Figure S2 presents the trade-off between aspect ratio *k* and estimation performance for λ = 0.01. We evaluated *k* = 2, 4, and 6 by simulating 50 noise realizations (note that $k_{\text{SERO}}$ can be any integer). The mean accuracy and precision (mean variance per voxel) of the estimated $T_{1}$ values in each sub-voxel were computed. Results indicate that *k* = 4 provided the most favorable accuracy versus precision trade-off: lower *k* (2) yields lower accuracy, whereas higher *k* (6) increases variability.


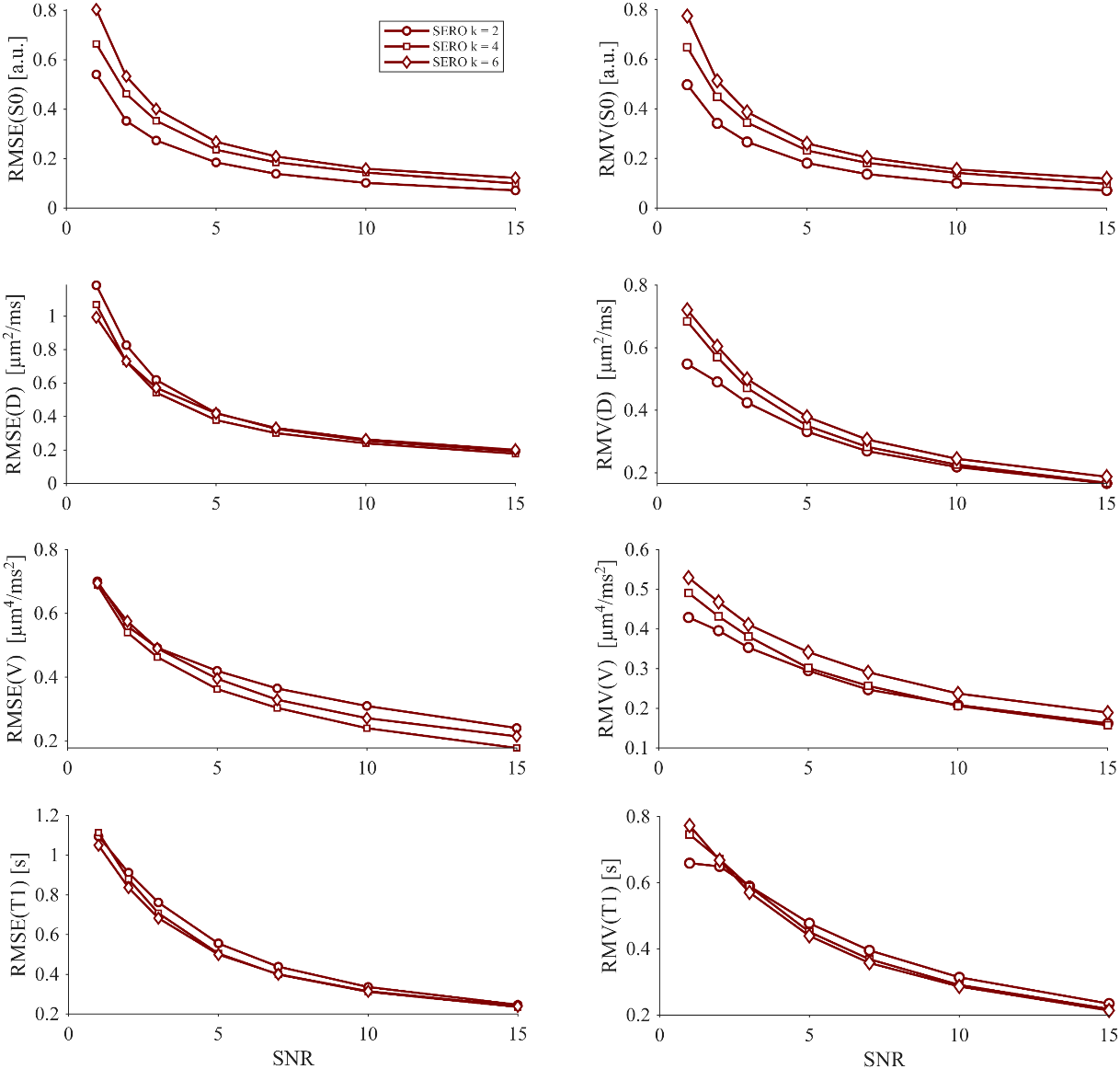


Figure S2 – Trade‑off between aspect ratio *k* and estimation performance at λ = 0.01. Curves show that *k* = 4 offered the best accuracy versus precision compromise: reducing to k = 2 increased the root-mean-squared-error (RMSE), whereas increasing to k = 6 raised voxel-wise root-mean-variance (RMV) levels. The intermediate *k* = 4 setting was therefore adopted for all other calculations.

### **4. Fisher-information Φ-criterion derivation**

A Fisher information analysis was included to quantify identifiability and the minimum achievable variance (Cramér–Rao bounds) for reconstructed parameters, clarifying why SERO improves estimation and guiding parameter-selection. Consider a thick imaging slice that can be regarded as two homogeneous sub-voxel segments stacked along the slice direction. Each sub-voxel has its own baseline signal $(S_{0j})$ and longitudinal relaxation time $(T_{1j})$ for *j* = 1, 2. If the two sub-voxels are sampled with separate repetition-time schedules TR_1_ and TR_2_ (each containing of *m* = 100 intervals from 0.16 to 16 s), the total measured signal is

|  | $S=S_{01}\left[ 1-\exp\left( -\frac{TR_{1}}{T_{11}} \right) \right]+S_{02}\left[ 1-\exp\left( -\frac{TR_{2}}{T_{12}} \right) \right].$ | (Eq. S3) |
| --- | --- | --- |

Stacking the $2m$ measurements into a vector **s** and linearizing the forward model around the true parameters $\boldsymbol{\theta} = {(S_{01}, T_{11}, S_{02}, T_{12})}^{T}$ yields $\mathbf{s}\boldsymbol{=}f\boldsymbol{(}\boldsymbol{\theta}) + \mathbf{n}$ with additive Gaussian noise $\boldsymbol{n \sim}\mathcal{N}(0, \nu_{n}\mathbf{I)}$ for sufficiently high SNR ratios^9^.

The Fisher sensitivity matrix $\boldsymbol{F \in}\mathbb{R}^{2m\times4}$ collects the partial derivatives ${df}/{d\theta}$ for every TR sample. Its information content is quantified by the D-optimality, or $\phi$-criterion^52^, with $p = 4$ such that

| $\phi(\mathbf{F}) = \det{(\mathbf{F}^{T}\mathbf{F})}^{1/p},$ | (Eq. S4) |
| --- | --- |

so that larger $\phi$ implies a lower Cramér-Rao variance bound for unbiased parameter estimates. Thus, larger values of $\phi$ imply smaller noise-induced variance of the identified parameters. In general, the $\phi(\mathbf{F})$ criterion takes different values within the parameters range^53^.

Suppose $S_{0,1}=1.0{a.u., T}_{1,1}=1.5 s, S_{0,2}=1.2 a.u.$and $T_{1,2}=1.0$ s. If both sub-voxels are driven by the same TR list (TR_1_ = TR_2_) the column of **F** almost becomes linearly dependent and the determinant is small, $\det{(\mathbf{F}^{T}\mathbf{F})}^{1/p} \approx9\cdot{10}^{-5}$, resulting in $\phi\approx0.17$ and a variance floor of around $1.6\times{10}^{4}\nu_{n}$.

In contrast, using two distinct lists, e.g. $\text{T}\text{R}_{\text{1}}=\{0.32, 0.64, \ldots, 16.0\}$ and $\text{T}\text{R}_{\text{2}} =\{0.16, 0.48, \ldots, 15.84\}$ raises the determinant by four orders of magnitude, to $\det{(\mathbf{F}^{T}\mathbf{F})}^{1/p} \sim1.49 \times{10}^{-1}$ so that $\phi\sim0.33$ and the bound falls to 64$\nu_{n}$. Hence, alternating the repetition-time schedules adds numerical precision.

Extending this framework to four sub-voxel segments entails modelling the signal as the sum of four contributions, such that

| $S=\sum_{j=1}^{4} S_{0j}\left[ 1-\exp\left( -\frac{TR_{j}}{T_{1j}} \right) \right],$ | (Eq. S5) |
| --- | --- |

which introduces $p = 8$ unknowns. A single common TR history renders **F** rank-deficient so that $\phi$ vanishes. Providing four distinct sets of TR for each sub-voxel segment restores full rank and a positive $\phi$, re-establishing a well-posed estimation problem.

In the SERO acquisition ($k = 4, n = 50$ target high-resolution voxels, $m = 1000$ shots, $p = 200$), each shot excites a slice composed of four segments. For every shot, the slice position is chosen at random anywhere inside the imaging volume rather than being moved in a fixed step. A high-resolution voxel therefore falls inside the excited slice whenever the center of the slice is within two voxels of its position. Given the parameters above, the chance of that happening on any one shot is 4 / 50 = 0.08, so each voxel is excited roughly 0.08 × 1000 ≈ 80 times over the full experiment, although the exact number varies from voxel to voxel. Every time the voxel is excited it contributes a separate row to the global Fisher matrix in the columns corresponding to that voxel’s parameters. Because these rows occur at different repetition times, the resulting matrix $\mathbf{F}^{T}\mathbf{F}$ stays well-conditioned and the $\phi$ criterion remains comfortably above zero throughout the typical parameter range, with only minor reduction at the edge of the FOV.

### **5. Residual-based SNR estimation for in-vivo acquisitions**

Because each signal acquisition in SERO samples a different TR, identical repetitions are unavailable precluding conventional SNR estimation. We therefore estimate SNR from the model-fit residuals.

Let $S_{i}\left( \mathbf{x} \right)$ be the observed signal for each shot *i* contributing to voxel *x*, and $\hat{S}_{i}\left( \mathbf{x} \right)$ the signal predicted by fitted model. For the set of contributing shots, we compute the standard deviation from the residuals

| $\hat{\sigma}\left( \mathbf{x} \right)=\sqrt{\frac{1}{n-1}\sum_{i=1}^{n} \left( S_{i}\left( \mathbf{x} \right)-\hat{S}_{i}\left( \mathbf{x} \right) \right)^{2}.}$ | (Eq. S6) |
| --- | --- |

An SNR map is then recovered as

| ${SNR}_{\mathrm{mean}}=\frac{\hat{S}_{i}(\mathbf{x})}{\hat{\sigma}\left( \mathbf{x} \right)}.$ | (Eq. S7) |
| --- | --- |

Reported SNR in vivo was the average SNR within a selected region of interest in the SNR map.
